# The Distribution and Cause of Cancer

**Published:** 1903-06-27

**Authors:** 


### Hospital Clinics and Medical Progress.

### THE DISTRIBUTION AND CAUSE OF CANCER.

Several interesting papers have appeared lately on the distribution and cause of cancer from the pen of Mr. D'Arcy Power, Dr. Brand, Dr. Neve, Dr. Arathoon, and Mons. J. Drivon. The writers have worked independently of each other by different methods and in different parts of the world, yet they are in agreement on several points. They all believe that heredity has little if any influence upon cancer, for there is no evidence that cancer has ever been transmitted. Children born of women suffering from cancer are not cancerous, nor is the mother of a child born with malignant disease herself affected, says Dr. Brand. All the writers agree that cancer is more common in rural than in urban districts when due allowance is made for the deaths which have taken place in the hospitals found in every large town. Statistics, however, are as misleading in this inquiry, as in others of a similar nature, but Mr. D'Arcy Power has been fortunate enough to obtain a record of all the deaths occurring during the last thirty-four years in a village with a population of 456 persons, and in this way some of the statistical fallacies have been avoided. Two hundred and sixty-four deaths are recorded as taking place in the village from 1869-1902, and of these, 27 were caused by phthisis and 17 by cancer. Of these two diseases cancer was the preponderating cause of death until 1902, but in this year there were seven cases of cancer in this small population, and by the end of the year five of the seven had died. The men in the village were affected with cancer as often as the women which is quite unusual. Mr. D'Arcy Power points out that the chief weakness in these figures is the smallness of the numbers dealt with, because it gives a wholly disproportionate value to a few additional cases of cancer which may occur, and in all probability did occur, quite accidentally. He further shows that the people dying in the village were either very old or very young, because many of the young and healthy had left their native village in search of work elsewhere. It is a matter of common knowledge that cancer is a disease of old people, and in a village population therefore consisting mainly of children and old people the cancer mortality is sure to be above the average. Mons. Drivon puts the same conclusion in a different way when he says that the increase of cancer is a product of higher civilisation and better hygiene. The statement is a paradox, but the higher the civilisation and the better the hygienic surroundings the greater is the average duration of life so that more persons survive to the time when cancer is common. The writers are in disagreement as to the cause of cancer. Dr. Brand is emphatic as to its infective nature, and maintains that the discovery of a causal micro-organism, fulfilling Koch's postulates, is only a matter of time. He would therefore make compulsory the notification of all cases of malignant disease that each case might afterwards be investigated thoroughly. Mr. D'Arcy Power is more guarded in his expression of opinion on the matter though he says that he has not quite lost faith in the parasitic theory of cancer. He also says that the more he sees of cancer cases the more struck is he by the frequency with which a widow or widower dies of malignant disease when the husband or wife has previously died of cancer. He thinks that these cases are more than mere coincidences and that they may point to some common factor in the joint lives of the sufferers which has allowed their decadent epithelial cells or connective tissue to run riot. The factor may be predisposing, as is the more likely, in which case it might be of a chemical nature associated with modern alterations in diet and habits; or it might be an exciting cause such as a parasite or other infective agent. Mons. Drivon is frankly agnostic, he says the only conclusion that can be drawn is that cancer is increasing, but no reason can be assigned for the increase. It is not due to an abuse of nitrogenous food as Verneuil taught; it is not infective.

There is no doubt, however, that injury is a fruitful antecedent of malignant disease, especially if the injury is followed by prolonged irritation. The teaching that phimosis predisposes to epithelioma of the penis receives confirmation in India on a large scale. The Mussulman population is very large, all its members are circumcised and they appear to be almost wholly free from cancer of the penis. The Hindoos, on the other hand, who have long foreskins and are negligent of cleanliness, suffer frequently. Cancer of the penis is one of their most common forms of cancer. Next in frequency to cancer of the penis comes cancer inside the mouth, but cancer of the lips is much more rare than it is in Europe, and the explanation is not far to seek. The smoking of pipes in India is comparatively rare, but the chewing of betel leaf with a mixture of lime and other ingredients is universal.

